# Evaluation of outpatient prescription patterns of COVID-19 drugs in Iran: comparison of real practice with local therapeutic recommendations

**DOI:** 10.1186/s40545-023-00672-8

**Published:** 2023-11-27

**Authors:** Amir H. Zargarzadeh, Zoha Abdi, Sarah Mousavi

**Affiliations:** 1 Department of Clinical Pharmacy and Pharmacy Practice, School of Pharmacy and Pharmaceutical Sciences, Isfahan University of Medical Sciences, Hezar Jerib Avenue, Isfahan, Iran; 2 School of Pharmacy and Pharmaceutical Sciences, Isfahan University of Medical Sciences, Isfahan, Iran

**Keywords:** Covid-19, Outpatient care, Drug prescription, Evidence-based medicine, Iran

## Abstract

**Background:**

A comprehensive guideline named “Diagnostic Therapeutic Flow Chart for Covid-19″ (DTFC) was released by the Scientific Committee of Covid-19 of Iran’s Ministry of Health and Medical Education and regularly was updated. The aim of this study was to compare the prescription pattern of drug treatment in outpatient Covid-19 patients with the DTFC.

**Methods:**

A cross-sectional study was conducted on the prescription pattern of drugs given to outpatients with a diagnosis of Covid-19, in Isfahan City from June to September 2021 (concurrent with the fifth peak of Covid-19 in Iran) taking into account the recommendations of the 9th version of DTFC (December 2020). A total of 8250 prescriptions were retrieved from the Public Health Department of Isfahan City.

**Results:**

Famotidine 20, 40 mg tablets (*N* = 936 patients) was the most prescribed drug followed by dexamethasone ampule (*N* = 588), prednisolone 5, 50 mg tablets (*N* = 478), azithromycin 250, 500 mg capsules (*N* = 452), diphenhydramine syrup (*N* = 362), vitamin D3 soft gel 50,000 Iu (*N* = 526), naproxen 250, 500 mg tablets (*N* = 266) and favipiravir 200 mg tablet (*N* = 191). The following drugs were administered against the recommendation of the DTFC-9: azithromycin, favipiravir, remdesivir, cetirizine, corticosteroids, vitamin C, vitamin B12, multivitamins, proton pump inhibitors (e.g., omperazole, anticoagulants (rivaroxaban,….), aspirin and ivermectin. Administration of analgesics, famotidine, hydroxychloroquine, vitamin D3, diphenhydramine and statins were in concordance with the DTFC-9.

**Conclusion:**

In this study, we showed frequent use of drugs with unproven efficacy in outpatient cases of Covid-19 mostly attributed to corticosteroids and antibiotics use. Our research highlights the discrepancy between recommendations for care and clinical practice and the need for strategy to bridge gaps in evidence-based informed decision-making.

## Introduction

From the February 19, 2020, the Iran officially announced the death of two patients due to Covid-19, more than 7.5 million cases were detected and 145 thousand deaths reported [1]. Iran is still one of the countries to deal with most cases of Covid-19 infection and subsequent deaths. From the first days of pandemic, Centers for Diseases Control and prevention (CDC) of Iran’s Ministry of Health and Medical Education (MOHME) established “Scientific Committee of Covid-19” for early detection and treatment of patients, risk management strategies, infection prevention, control strategies and national instructions [2]. The committee released a comprehensive guideline named “Diagnostic and Therapeutic Flowchart for Covid-19” abbreviated as DTFC and regularly was updated with several changes according to the national and international experience [3]. The flowchart consists of several parts including active clinical screening, diagnostic tools, outpatient and inpatient care of mild, moderate and severe cases of Covid-19.

The screening in Iran has mainly been clinically oriented and symptoms based. As part of screening, MOHME launched a short web-based questionnaire, through which people received a notification of having possibly Covid-19 and were asked to go to the nearest health care center for follow-up and management. However, the polymerase chain reaction (RT-PCR kits) were reserved for suspected cases [4].

For outpatient care of Covid-19 cases, the DTFCs strongly recommended home care in all patients upon exacerbation of symptoms such as dyspnea, loss of consciousness, continuation of fever and relentless cough. All versions of DTFC emphasized that Covid-19 should be managed in most patients without special antiviral or antibiotic therapies and the adjunctive medications were restricted to febrile high-risk patients and immunocompromised with normal lung imaging [5].

The DTFC-9 which was addressed in this study proposed hydroxychloroquine sulphate 200 mg two tablets twice daily on the 1st day and then one tablet twice daily for 7 days. Other recommendations were as follows: (1) enough rest, appropriate meal, high fluid intake and isolation; (2) analgesics: acetaminophen as 1st line and if the patient not responsive, NSAIDs could be replaced (naproxen due to favorable safety profile as first line). 3) Anti-tussive medication, bromhexine, dextromethorphan, diphenhydramine and some approved herbal products; (4) anti-nausea medication such as dimenhydrinate; (5) ORS and loperamide in case of diarrhea; (6) in case of deficiency, vitamin D3 pearl (50,000 IU/week for 8–12 weeks), zinc and selenium; (7) famotidine as antacid. Also, the guideline strongly recommends against the routine use of the following medications: (1) anticoagulants, (2) aspirin, (3) azithromycin, (4) doxycycline, (5) steroids, (6) proton pump inhibitors (e.g., omeprazole, pantoprazole), (7) unapproved herbal products and (8) multivitamins [5].

Delta variant of COVID-19 started in June 2021 in Iran and led to huge increase in the number of cases and deaths which was called the fifth peak of COVID-19. The sudden increase in the number of cases led to a shortage of hospital beds, medications and overwhelming of medical staff [6]. The MOHME emphasized that the outpatient care of Covid-19 should be observed using DTFCs recommendations. So, the aim of this study was to compare the prescription pattern of drug treatment in outpatient Covid-19-positive patients with the recommendations issued by MOHME through the DTFC guideline during the fifth peak.

## Methods

Isfahan, with a land area of 43,978 km is the capital of Isfahan Province and has a population of approximately 2,220,000 making it the third largest city in Iran.

A cross-sectional study was conducted on the prescription pattern of drugs given to outpatients with a diagnosis of Covid-19, in Isfahan City from June to September 2021 (concurrent with the fifth peak of Covid-19 disease in Iran). The profile of patients with positive PCR test due to Covid-19 disease was inquired from the Vice-Chancellor of Health of Isfahan University of Medical Sciences. According to the national protocol (DTFC9), symptomatic and suspicious patients who needed a preliminary and accurate diagnosis of Covid-19, were referred to 16-h centers and PCR tests were taken from them if necessary. In the city of Isfahan, 17 centers provided services to these patients 16 h a day. Therefore, the details of the PCR positive patients caused by Covid-19 from these centers were requested by the health department for the period from June to September 1400. Based on the information obtained (National Patient Code) from insurance companies, i.e., Tamin Ejtemaie and Salamat for the patients who had an outpatient visit and were infected with Covid-19, electronic prescriptions were taken and analyzed. In addition, each national code was evaluated once.

A data base in Microsoft Excel 2022 was set up to record the information on the drugs in the prescriptions including: name of the drug, dosage form and strength, number of drugs per patient and total number of drugs. The obtained data were compared with DTFC-9 (December 2021) guideline [6] in terms of the types of prescription drugs and observance of the recommendations of the guideline about outpatient care of Covid-19 cases. Also, prescribed drugs were checked for drug–drug interaction using Lexicomp version 2022 drug interaction checker. Lexicomp is a medical software which gives evidence-based drug information about DDIs. It categorizes drug combinations into X (contraindicated), D (consider therapy modification), C (monitor therapy) B and A (no action needed).

The analysis was performed by Microsoft Excel software. Results have been reported as frequency and percentage.

## Results

A total of 8250 prescriptions were identified. The minimum and maximum number of drugs prescribed to a single patient was 1 and 10, respectively. The average number of drug per prescription was 4.9 ± 3.1. Most of the prescriptions (*N* = 1352) had two drugs.

Among the 8250 prescriptions evaluated, famotidine (*N* = 936 patients, 18,954 total number of prescribed drug), was the most prescribed drug followed by dexamethasone (*N* = 588, 2385), normal saline (*N* = 528, 1060), prednisolone (*N* = 362, 6571), azithromycin (*N* = 452, 3279), diphenhydramine (*N* = 362, 379), vitamin D3 50,000 IU soft gel (*N* = 256, 2153), naproxen (*N* = 266, 4050), favipiravir (*N* = 191, 7496) and cetirizine (*N* = 187, 1570). Table 1 shows the five most prescribed drug among different categories.

**Table 1:** Frequency of drugs prescribed

Drug class	Drug name	Dosage form and strength	Number of drug per patients	Total number of prescriptions
Analgesics	Naproxen	Tablet 250 mg	138	2475
	Naproxen	Tablet 500 mg	128	2030
	Acetaminophen	Tablet 500 mg	173	3450
	Ketorolac	Ampule 30 mg	182	273
	Diclofenac sodium	Suppository 100 mg	82	623
	Piroxicam	Ampule 20 mg	51	84
Antibiotics/antivirals	Azithromycin	Tablet 250 mg	121	1018
	Azithromycin	Tablet 500 mg	331	2261
	Favipiravir	Tablet 200 mg	191	7496
	Hydroxychloroquine	Tablet 200 mg	185	3126
	Cefixime	Tablet 200 and 400 mg	133	1498
	Remdesivir	Ampule 100 mg	115	545
Antihistamines/cough suppressants	Diphenhydramine	Syrup	362	379
	Chlorpheniramine	Ampule 10 mg	134	230
	Cetirizine	Tablet 5 and 10 mg	115	1570
	Theophylline G	Syrup	90	93
	Bromhexine	Syrup	90	95
	Montelukast	Tablet 10 mg	84	1381
Anti-inflammatory	Dexamethasone	Ampule 8 mg/ml	566	2045
	Prednisolone	Tablet 5 and 50 mg	478	6571
	Hydrocortisone	Ampule 100 mg	108	220
	Methylprednisolone acetate	Ampule 40 mg	64	109
	Betamethasone disodium phosphate	Ampule 4 mg/ml	58	102
	Fluticasone/salmeterol	Spray 250/50 mcg	64	64
Vitamins and minerals	Vitamin D3	Soft gel capsule 50,000 Iu	256	2153
	Vitamin D3	Ampule 3,000,000 Iu	112	163
	Vitamin B1	Tablet 300 mg	95	2610
	Vitamin B12	Ampule	80	148
	Zinc sulphate	Capsule 220 mg	76	1280
	Vitamin B complex	Ampule	69	101
Gastrointestinal drugs	Famotidine	Tablet 20 mg	148	3285
	Famotidine	Tablet 40 mg	788	15,669
	Ondansetron	Ampule 2mg	58	154
	Ondansetron	Tablet 4 mg	39	665
	Omeprazole	Capsule 20 mg	49	1352
	Clidinium C	Tablet	46	1530
Cardiovascular drugs	Aspirin	Tablet 80 mg	132	5585
	Atorvastatin	Tablet 10 and 20 and 40 mg	102	5680
	Enoxaparin	Ampule 4000 and 6000 and 8000 Iu	46	333
	Losartan potassium	Tablet 25 and 50 mg	34	2680
	Rivaroxaban	Tablet 10 and 15 mg	32	617
Serums	Normal saline 0.9%	500 and 1000 ml	528	1060
	Dextrose/saline 1/3 2/3	500 and 1000 ml	311	432
	Dextrose/ saline 5%/0.9%	500 and 1000 ml	40	61
	Ringer	500 and 1000 ml	29	47
Other				
	Chlordiazepoxide	Tablet 5 and 10 mg	54	1579
	Gabapentin	Capsule 100 and 300 mg	64	2210
	Sertraline	Tablet 50 and 100 mg	36	1478
	Alprazolam	Tablet 0.5 and 1 mg	28	745
	Aminophylline	Ampule 250 mg	43	54

As shown in Table 1, the most prescribed antibiotics were azithromycin 500 mg, azithromycin 250 mg and amoxicillin 500 mg, favipiravir 200 mg, hydroxychloroquine 200 mg, cefixime 400 mg, ciprofloxacin 500 mg, respectively, none of which comply with the recommendations of DTFC-9 except hydroxychloroquine.

Among the anti-cough and antihistamine drugs prescribed for the treatment of Covid-19 outpatients, the following drugs comply with the recommendations of DTFC-9: Timex Plus, Tyman, Bromhexine, Broncobarij, Brenkeldin, Broncocold, Dextromethorphan, Dextromethorphan P, Thymus, Honeytos Diphenhydramine and diphenhydramine compound.

The highest amount of prescribed syrup was: diphenhydramine compound, theophylline G, bromhexine, diphenhydramine and the highest amount of tablets prescribed: chlorpheniramine 10 mg, cetirizine 10 mg, montelukast 10 mg and bromhexine 8 mg.

The highest number of prescribed inhalers among prescribed anti-inflammatory drugs was salmeterol/fluticasone 250/25. Among the prescribed anti-inflammatory drugs, the highest ampoule amount was as follow: dexamethasone, hydrocortisone, methylprednisolone, and betamethasone disodium phosphate.

Between the supplements and multivitamins prescribed for the treatment of outpatients with Covid-19, the supplements that comply with the recommendations of DTFC-9 include: vitamin D, zinc plus capsules, zinc and vitamin C chewable tablets and capsules, selenium plus, zinc plus, zinc sulfate.

Among the gastrointestinal drugs, the most prescribed tablets were: famotidine 40 mg, famotidine 20 mg, clidinium C, ondansetron and pantoprazole. Ondansetron and metoclopramide were the most prescribed ampules and omeprazole were the most prescribed capsules.

Among the drug categories, the medication used for the management of outpatients of Covid-19 through study period were most often anti-inflammatory and immunomodulators (*N* = 1632) (e.g., corticosteroids), antibiotics and antivirals (*N* = 1519), gastrointestinal drugs (*N* = 1492), antihistamine and cough suppressants (*N* = 1445), intravascular solutions (*N* = 1036) and analgesics (*N* = 1052).

Following drugs were administered against the recommendations of DTFC-9 for outpatient care of Covid-19: azithromycin, doxycycline, favipiravir, remdesivir, corticosteroids (dexamethasone, prednisolone, methylprednisolone), cetirizine, hydroxyzine, montelukast, promethazine, vitamin C, calcium products, magnesium, vitamin E, multivitamins, vitamin B12, vitamin B6, vitamin B1, dicyclomine, diphenoxylate, PPIs, ondansetron, aspirin, anticoagulants (enoxaparin, heparin, rivaroxaban) and ivermectin.

Administration of analgesics (acetaminophen, NSAIDS), hydroxychloroquine, bromhexine, dextromethorphan, diphenhydramine, dimenhydrinate, some herbal products (Thymex^®^ syrup, containing Thyme for cough), vitamin D3, zinc, selenium, famotidine, ORS, statins and chlordiazepoxide were according to recommendations of DTFC-9.

Total of 158 drug–drug interactions were identified, from them 112 belonged to class C, 18 to class D, 19 to class X, 9 to class B. The most common class X interactions were as follows: naproxen and ketorolac, hydroxychloroquine and ondansetron, cyclosporine and atorvastatin, ibuprofen and ketorolac, diclofenac and ketorolac, azithromycin and hydroxychloroquine, and atorvastatin and gemfibrozil. Examples of class D were aspirin and naproxen, and dimenhydrinate and oxycodone. Examples of class C were cetirizine and chlordiazepoxide, azithromycin and diphenhydramine, enoxaparin and naproxen diphenhydramine and loratadine, cetirizine and diphenhydramine, and diphenhydramine and ondansetron.

## Discussion

The results of this study shows that despite recommendations of DTFC-9, most of the outpatients cases of Covid-19 were managed with therapies which did not correlate with the guideline and were without documented efficacy or indications. Although the DTFC emphasized that most of the Covid-19 outpatients should be managed without special antiviral or antibiotic therapies, nearly, all patients received an antibiotic (mostly azithromycin or doxycycline) and antivirals (remdesivir and favipiravir).

The fifth peak of Covid-19 in Iran (started June 2021 with delta variant) led to the sudden increase in the number of cases and shortage of hospital beds [6]. Despite the obligations of physicians to prescribe such therapies, it has been described that in time of crisis such as Covid-19 pandemic, clinicians are inclined to prescribe unproven therapies, which may be potentially harmful. Our results confirm that with a high percentage of class X drug–drug interactions (combination of hydroxychloroquine and azithromycin and ondansetron) potentially could lead to QTc prolongation [6].

The prescription of dexamethasone in the studied outpatients was very high, although its efficacy is mainly for severe Covid-19 [7] and was against DTFC recommendations.

Even though international guidelines such as WHO [8], recommend against the use of hydroxychloroquine (because of lack of efficacy), but due to controversial results for the available options (such as remdesivir, favipiravir) in the time of guideline development (2020) [9], DTFC-9 considered hydroxychloroquine sulphate for the treatment of outpatients with Covid-19 diagnosis. Nonetheless, as the international new evidence came out and our national guidelines fell behind the new evidence, it is interesting that our physicians did not prescribe hydroxychloroquine as the first line option and preferred other therapies as if they were more up to date than the DTFC-9.

The most used outpatient therapy was based on corticosteroids (dexamethasone, prednisolone) and antibiotic drugs especially azithromycin, despite the recommendations of DTFC-9. The increasing prescription of off-label drugs with unproven efficacy has been documented since the first months of the pandemic [10], where the prescription of azithromycin and hydroxychloroquine increased up to 195% and 1977% [10, 11], respectively. However, in other countries after the announcement of WHO regarding the lack of efficacy of hydroxychloroquine, there was a clear trend of decrease in the number of prescriptions [12] but not in Iran.

Fonts-Gonzales et al. [13] described the use of drugs in ambulatory patients with confirmed Covid-19 (*N* = 350) in Mexico City. There was ambulatory medical prescription in 172 (49%) patients. The prescription rate was high for hydroxychloroquine/azithromycin (19%) and dexamethasone (25%). The results were in concordance with ours. Belleudi et al. [12] recorded the use of outpatient drug treatments in Covid-19 positive population, taking into account the Italian regulatory agency’s advice in Lazio region, Italy. The use of drug therapy in the management of outpatient Covid-19 cases was frequent (one third of the cases). The most used drug therapy was antibiotics, especially azithromycin in spite of the recommendations of the regulatory agency. The use of hydroxychloroquine was limited to the early pandemic period and then reduced. The use of corticosteroids increased after the positive recommendations of the regulatory agency. Antithrombotics were widely used in outpatient settings despite its recommendations of its use only in hospitalized patients. In our study, we found high rates of dexamethasone and anticoagulants administration opposite to the DTFC-9 recommendations.

The most prescribed drug was famotidine, although its prescription is in concordance with DTFC-9, but, it has unproven efficacy [14]. The DTFC-9 recommends vitamin D3, zinc and selenium in concordance with the first recommendations of WHO. The vitamin D3 was the most prescribed, although further evidence showed lack of its efficacy [15]. Administration of Vitamins B12, B1, C and B complex was high, all proved ineffective [16].

The analgesics prescription was in concordance with the recommendations of DTFC-9. But, most of the prescriptions contained acetaminophen in addition to a NSAID, which was considered as class X of drug–drug interactions. In the time of the fifth surge of Covid-19 in Iran, the paper prescriptions turned to electronic prescriptions. But, there were no drug–drug interaction software to alarm the physicians. Therefore, the high rate of class D and X of drug–drug interactions is substantial and need to be focused and addressed.

### Limitations of the study

Out-of-pocket expenses might have influenced on the proportion of prescription of study drugs, particularly for NSAIDs and vitamins. Also, due to some national security matters, we could not access the demographics of patients (age and sex) and their outcomes. Finally, the indication for drug prescription is not known, so, in some cases, the drug use could not be related to Covid-19 infection.

## Conclusion

Our study shows a frequent use of drug therapy in the management of outpatient cases of Covid-19, mainly corticosteroids and antibiotics. Our research highlights the discrepancy between recommendation of DTFC-9 and clinical practice of the physicians and the need for strategies to bridge the gap. Off-label drug use without any control or appropriate protocol represents a health risk that can be avoidable. The regulatory bodies should find new ways to promptly respond to the uncertainty with experimental treatments based on spurious data. Correct and evidence-based information on the appropriate use of drugs is crucial in a context of high uncertainty such as pandemic.

## Data Availability

The authors confirm that the data supporting the findings of this study are available within the article.
